# Advances in polycythemia vera treatment with targeted therapies and clinical trials

**DOI:** 10.1007/s12672-025-03703-9

**Published:** 2025-10-27

**Authors:** Doaa Hassanien, Rashid A. AlYafei, Omar H. Metwally, Izzaldin Alremawi, Abdulla Khalid Al-Kaabi, Mohamed Elahtem, Talal AL-Sayed, Mohamed A. Yassin

**Affiliations:** 1 Neurology Department, Hamad Medical Corporation (HMC), P. O. Box 3050, Doha, Qatar; 2 College of Medicine, QU Health, Qatar University, P. O. Box 2713, Doha, Qatar; 3 Internal Medicine Department, Hamad Medical Corporation, Doha, Qatar; 4 Hematology Section, Medical Oncology, National Center for Cancer Care and Research (NCCCR), Doha, Qatar

**Keywords:** Polycythemia vera (PV), Chronic myeloproliferative disorder (CMD), Hepcidin agonists, MDM2 inhibitors, Histone deacetylase (HDAC) inhibitors, LSD1 inhibitors, Clinical trials

## Abstract

Polycythemia vera (PV) is a chronic myeloproliferative disorder characterized by excessive red blood cell production, leading to a heightened risk of thrombosis, hemorrhage, and progression to myelofibrosis. While traditional therapies such as phlebotomy and hydroxyurea have been used for disease management, they do not address the underlying pathophysiology or alleviate common symptoms like fatigue and pruritus. Recent advances in targeted therapies offer promising new options for PV treatment. This review explores novel therapeutic approaches currently under investigation, including hepcidin agonists, MDM2 inhibitors, histone deacetylase (HDAC) inhibitors, and LSD1 inhibitors. These therapies aim to correct hematopoietic dysregulation, reduce symptom burden, and improve long-term outcomes for PV patients. While clinical trials show encouraging results, further studies are needed to fully evaluate their safety, efficacy, and potential for broad clinical use. Ultimately, these emerging treatments could reshape the landscape of PV management by offering more personalized, effective options for patients.

## Introduction

### Overview of polycythemia

Polycythemia vera (PV), essential thrombocytosis, and primary myelofibrosis are classified as the main types of BCR-ABL-negative myeloproliferative neoplasms (MPNs) [1, 2]. These are a group of diverse blood cancers caused clonal mutations in JAK1 and JAK2 in hematopoietic stem cells, leading to persistently active signaling pathways [2, 3]. PV is defined by an excess of red blood cells, an overactive bone marrow, fatigue, microvascular symptoms, and an enlarged spleen [2]. PV is associated with significant complications, including a markedly increased risk of thrombosis (41%) [4]. Additional challenges include poor quality of life and potential progression to myelofibrosis or MPN-blast phase. Patients with PV experience burdensome symptoms such as fatigue, pruritus, concentration problems, and cognitive impairment, often linked to anemia [5]. Median survival rate of PV was 23 years in patients without risk factors and 9 years in patients with risk factors [6]. Treatment goals in PV focus on relieving symptoms, lowering thrombosis risk, and preventing progression to myelofibrosis or MPN-blast phase [7].

### Risk stratification and management

Cardiovascular events are the major cause of death in PV patients with thrombosis being the main cause of death [4]. The two most common predictors of cardiovascular events are age more than 65 and history of thrombosis [8]. Based on these factors, PV patients are categorized into low- and high-risk groups, which guide their management plans. For low-risk patients, standard treatment includes phlebotomy to maintain a hematocrit level below 45% in both males and females, combined with daily low-dose aspirin (40–100 mg) [9]. If microvascular symptoms, leukocytosis, or cardiovascular symptoms persist, the aspirin dose can be increased to twice daily [8]. High-risk patients, defined as those older than 60 years or with a thrombosis history, are typically prescribed cytoreductive therapy, starting with hydroxyurea (HU) at an initial dose of 500 mg twice daily [9]. These patients should also receive twice-daily aspirin and systemic anticoagulation if they have a history of arterial or venous thrombosis [10]. For the 11–24% of patients who develop HU resistance or intolerance, alternative treatments such as pegylated IFN-alpha or busulfan are recommended [11]. These therapies provide additional options for managing high-risk patients and improving outcomes.

### Challenges with standard therapies

Although phlebotomy and cytoreductive agents are standard therapies, their frequent use has contributed to the disease burden. Fatigue, the most commonly reported complaint, is often associated with anxiety, worry, and worsening symptoms such as pruritus. Together, these factors result in a poor quality of life [5, 12]. The REVEAL study reported that 865 patients experienced significant life-disabling complications following phlebotomy, including fatigue, bruising, dizziness, and dehydration, as well as long-term complications such as iron deficiency anemia. Additionally, iron supplementation exacerbates erythrocytosis in PV patients [13]. Standard therapy complications were also notable, including excessive bleeding with aspirin and systemic anticoagulation, myelosuppression, peripheral neuropathy, and face and mouth ulcers with HU, as well as basal and squamous skin cancer associated with long-term HU use [14].

### Interferons in PV

Given these concerns, HU is not recommended as a concurrent or alternative medication alongside phlebotomy in younger patients who may require prolonged HU exposure, as outlined by the European LeukemiaNet (ELN) 2021 recommendations [7]. Instead, pegylated interferon is recommended by both ELN and NCCN guidelines for younger patients [9, 15]. A Phase III clinical trial comparing pegylated interferon-α (PEG) with HU reported that both treatments had equal efficacy in terms of thrombosis rates and disease progression [16]. However, PEG was superior in normalizing counts and reducing JAK2V617F variant allele frequency (VAF), although grade 3/4 adverse events were more frequent with PEG [16]. Moreover, a 2024 meta-analysis suggests that ELN/NCCN recommendations may result in the undertreatment of PV patients under 60 years of age, as there is no clear evidence to support concerns that the risk of toxicity exceeds the potential benefit in reducing the risk of secondary myelofibrosis (sMF), myelodysplastic syndrome/AML, or death [17]. It’s Important to mention that Pegasys (Pegylated interferon-α2a) was withdrawn from the market.

### Established and emerging therapies

Ropeginterferon alfa-2b has long-term efficacy, demonstrable molecular activity, and a guideline-supported role, warranting more detailed discussion [18–20]. In the 6-year PROUD-PV/CONTINUATION-PV follow-up, patients on ropeg had higher event-free survival than control (0.94 vs 0.82; HR 0.34, 95% CI 0.12–0.97; p = 0.04), greater and more sustained hematologic control (more time spent in complete hematologic response and more stable leukocyte counts), and a marked reduction in JAK2V617F allele burden (median 8.5% vs 50.4% at month 72; ELN-defined molecular response 66.0% vs 19.4%) signals consistent with disease modification [21]. In the randomized phase-3 analysis, hematologic responses improved over time vs hydroxyurea (e.g., CHR 71% vs 51% at 36 months without the spleen criterion) and the safety profile differed qualitatively more liver-enzyme elevations with ropeg, more cytopenias with hydroxyurea; serious treatment-related adverse events were low in both arms, with one treatment-related death in the standard-therapy arm [21]. Ropeginterferon alfa-2b also provides practical dosing (q2–4 weeks) and is EMA approved (2019) and FDA-approved (2021) for PV, regardless of risk category. Finally, current guidance notes its role in treatment algorithms: the ELN recommends ropeg for low-risk PV patients who require cytoreduction, and the NCCN lists ropeg as a preferred option when initiating cytoreductive therapy; supportive data include LOW-PV, where a fixed ropeg dose outperformed phlebotomy for maintaining hematocrit ≤ 45% without disease progression [18, 20, 21].

Beyond RPG, other innovative therapies are also gaining attention. Both the CONTINUATION-PV trial and the MAJIC-PV trial, which investigated ruxolitinib, provided evidence of their ability to reduce disease burden and potentially modify disease progression [21, 22]. A growing number of novel treatments targeting diverse pathways have emerged as promising options for managing erythrocytosis in PV. These therapies aim to shift the focus from hematocrit control toward comprehensive disease modification [23, 24]. Furthermore, there is increasing emphasis on improving patients' quality of life by alleviating common side effects of standard therapies, including hospital dependency, fatigue, pruritus, and anemia [5, 13, 25]. This paper will review the novel therapeutic approaches for PV that aim to modify the natural progression of the disease, with a focus on the safety and efficacy of these treatments as demonstrated in different phases of preclinical and clinical trials (see Tables 1 and 2).

**Table 1:**
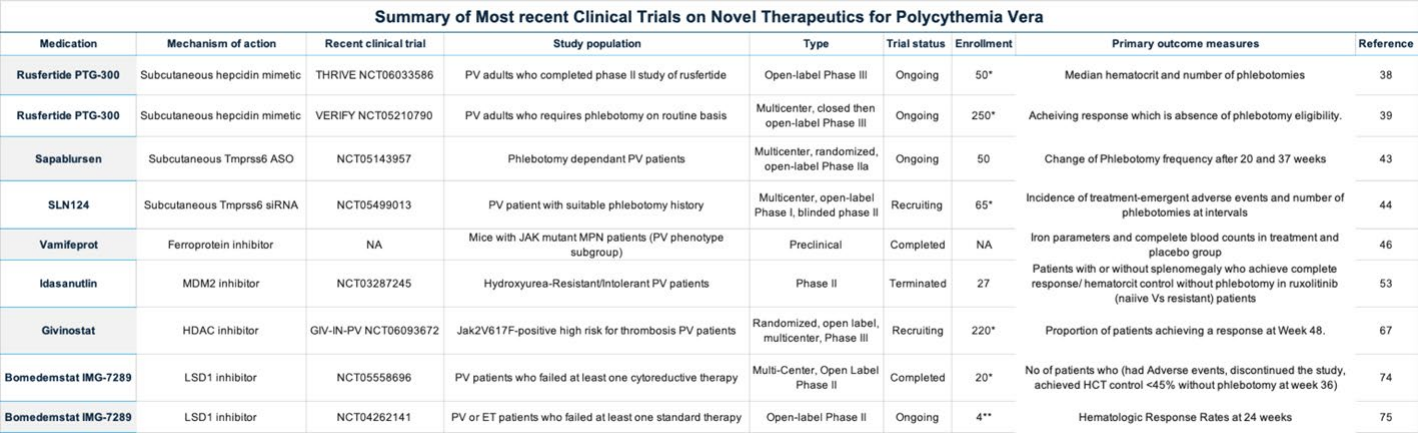
Summary of the most recent clinical trials on novel therapeutics for polycythemia vera

*This is the estimated not the actual recruited number of patients

**Table 2:**
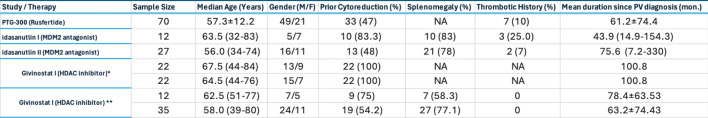
Demographics and baseline characteristics of patients in phase II clinical trials of novel therapies for polycythemia vera

*Givinostat was given to two groups at 50mg and 100mg doses

## Hepcidin agonists

### Background and therapeutic rational

**Fig. 1:**
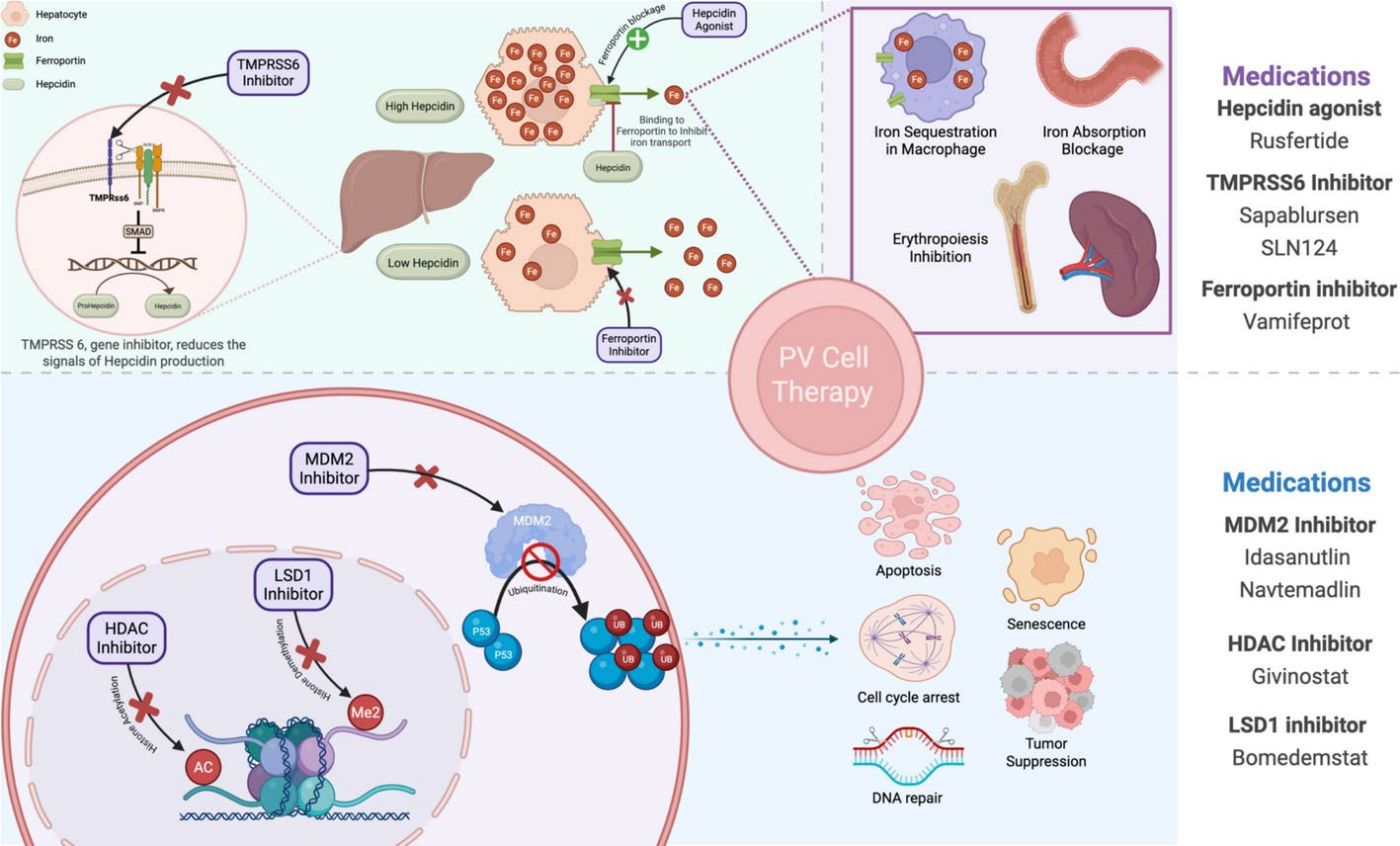
Novel treatment approches to polycythemia vera and their mechanism of action. Created in BioRender. Hassanein D. 2025. https://BioRender.com/dpr6pp1

Hepcidin, a peptide hormone mainly produced by liver cells, plays a central role in regulating iron balance in the body (Fig. 1) [26]. It controls dietary iron absorption, iron recycling by macrophages, and iron release from liver stores [26]. Hepcidin limits the release of iron into the bloodstream by binding to ferroportin 1, the only known iron exporter, effectively reducing its function [27]. When hepcidin levels are high, iron absorption is blocked, and iron becomes trapped within hepatocytes and macrophages [26]. Conversely, low hepcidin levels allow for increased iron absorption and release from cellular stores, aiding in the recovery from iron deficiency or addressing iron overload [28]. Pathological low levels of hepcidin are observed in iron loading anemia to suppress hepcidin and promote erythropoiesis, a process that is undesirable in PV patients [29]. Pharmacological trials of several hepcidin agonists have been developed in recent years as iron-restrictive therapies for PV, aiming to increase hepcidin levels, reduce iron and transferrin saturation, and inhibit erythropoiesis [30]. Hepcidin agonists are classified into hepcidin mimetics (e.g., Rusfertide), stimulators of hepcidin production (e.g., Sapablursen and SLN124), and ferroprotein inhibitors (e.g., Vamifeprot), which are used to treat various iron dysregulation disorders, including polycythemia vera, thalassemia, hemochromatosis, and anemia of chronic disease [30, 31].

### Rusfertide in PV (PTG-300)

Rusfertide [32]is a synthetic injectable hepcidin mimetics that works as an endogenous hepcidin. In preclinical trial on PV mice, hepcidin mimetics showed a significant hematocrit control < 45% eliminating the need for phlebotomy without causing iron depletion [33]. In the Phase 1 study, Rusfertide was well-tolerated with no severe or serious adverse events, and it showed dose-dependent reductions in serum iron and transferrin saturation (TSAT), with increased serum ferritin levels reflecting effective iron sequestration [34].

The PACIFIC trial (Phase II, NCT04767802) highlighted Rusfertide ability to rapidly reduce hematocrit (HCT) from an average of 51% to below 45% within six weeks, maintaining this level with weekly maintenance doses. Treatment interruptions led to increased HCT, therapeutic phlebotomy (TP) needs, and reduced ferritin levels, but these normalized when Rusfertide resumed, underscoring its critical role in hematologic control. Mild injection site reactions (59%) were the most common adverse events [32]. In the REVIVE trial (Phase II, NCT04057040), Rusfertide reduced annualized phlebotomy rates from 8.7 ± 2.9 to 0.6 ± 1.0 during a 28-week dose-finding phase, maintaining mean maximum HCT at 44.5% compared to 50.0% pre-treatment. During the randomized withdrawal phase, 60% of patients on Rusfertide achieved composite HCT control without phlebotomy, compared to 17% in the placebo group (P = 0.002). Patient-reported outcomes improved significantly, with reductions in symptoms like fatigue, pruritus, and night sweats. Serum ferritin levels increased consistently, aligning with Rusfertide’s mechanism of iron sequestration. Adverse events were primarily mild to moderate, with injection-site reactions being most common (Grade 3 events in 13% of patients and no Grade 4/5 events) (See Tables 3 and 4) [35, 36].

**Table 3:**
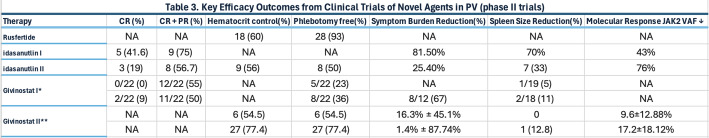
Key Efficacy Outcomes from Phase II Clinical Trials of Novel Agents in PV

*Givinostat was given to two groups at 50mg and 100mg doses

**Table 4:**
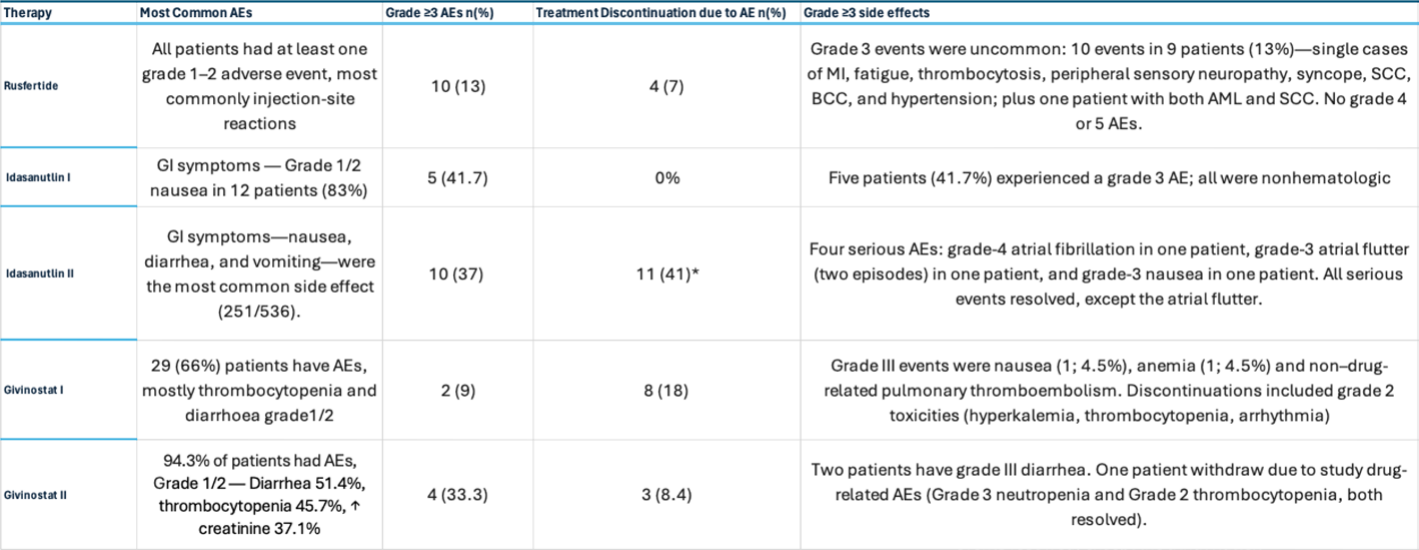
Safety and tolerability profiles of investigational therapies in PV

*Early study dropouts were largely attributed to non-severe gastrointestinal adverse effects, according to the investigation

Two ongoing Phase III clinical trials, VERIFY (NCT05210790) and THRIVE (NCT06033586), are investigating the long term efficacy of Rusfertide as a treatment for PV [37]. VERIFY is evaluating its safety and efficacy in controlling hematocrit and reducing the need for phlebotomies through a 32-week double-blind phase followed by a 124-week open-label extension, with completion expected by mid-2025 [38]. With the ongoing Phase III clinical trials, Rusfertide could become a viable first-line treatment.

### Sapablursen and SLN124

TMPRSS6 is a gene that encodes matriptase-2, a transmembrane protein that negatively regulates hepcidin production through BMP/SMAD signaling [39]. Reduced hepcidin levels facilitate iron absorption and the release of iron from stores, thereby increasing iron availability [39]. TMPRSS6 inhibitors are employed as iron-restrictive therapies in hematological disorders to elevate hepcidin levels that reduce iron and transferrin saturation, and inhibit erythropoiesis [39]. In PV mice, administration of a TMPRSS6-ASO effectively reversed erythrocytosis and normalized hematocrit levels. These findings suggest that TMPRSS6-ASO therapy could act as a “medical phlebotomy”, providing ongoing control of accelerated erythropoiesis in PV treatment [39, 40].

SLN124, a short interfering RNA (siRNA) targeting TMPRSS6 mRNA, increases hepcidin transcription. A Phase I clinical trial of SLN124 was conducted in 24 healthy individuals, demonstrating that SLN124 was well tolerated, with no severe or serious adverse events [41]. SLN124 exhibited a dose-dependent effect, increasing plasma hepcidin levels (peaking at Day 29) and reducing serum iron, transferrin saturation, reticulocyte production, MCHC, and MCV, with effects lasting up to 56 days [41]. Mild injection site reactions were the most reported side effects, however, three participants discontinued due to sustained low transferrin saturation (< 10%), as per protocol guidelines. Overall, Sapablursen was safe, well tolerated, and had a pharmacokinetic half-life of 2–4 weeks [41]. Currently, 40 PV participants are enrolled in an ongoing Phase IIa clinical trial (NCT05143957), a multicenter, randomized, open-label study assessing the efficacy of Sapablursen in reducing phlebotomy frequency and improving quality-of-life measures [42]. In addition, a Phase 1/2 multicenter study is ongoing to further assess the safety, tolerability, efficacy, pharmacokinetics (PK), and pharmacodynamics (PD) of SLN124 in PV patients [43].

### Vamifeprot

Vamifeprot is a ferroprotein inhibitor that blocks the release of iron into the bloodstream, thereby reducing iron availability [44]. It is being explored as a potential therapy for conditions associated with iron dysregulation, such as thalassemia and sickle cell anemia where it has demonstrated significant safety and efficacy [44]. In JAK2-mutant mouse models of PV, ferroprotein inhibitors effectively normalized hematocrit and hemoglobin levels, underscoring their potential as promising therapeutic options for PV patients [45].

## MDM2 inhibitor

### Background and therapeutic rational

In 1987, the MDM2 gene was discovered, and since then, numerous studies have explored its oncogenic effects, pathophysiological mechanisms, and potential therapeutic approaches [46]. Clinical studies have identified MDM2 amplification and overexpression in various cancers, including PV, where MDM2 levels are notably higher in PV patients compared to healthy individuals [48]. MDM2 downregulates the tumor suppressor p53 through its E3 ligase activity, and overexpression of MDM2, driven by various genetic mutations, leads to reduced p53 tumor suppression [48, 49]. In vitro studies have shown that combining MDM2 antagonists with Peg IFN-α 2a effectively targets hematopoietic progenitor cells in PV, significantly reducing the population of malignant progenitor cells (PV) [49]. Despite this promising preclinical evidence, no MDM2 antagonist has been FDA-approved yet, although several have been evaluated in human clinical trials [50]. This section reviews the clinical trials involving different MDM2 antagonists for PV patients.

### Idasanutlin in PV

In Phase I clinical trial (NCT02407080), Idasanutlin demonstrated significant clinical activity with an overall response rate (ORR) of 58% for monotherapy and 50% for combination therapy with PEG, resulting in a combined ORR of 75% [51]. Median JAK2 V617F allele burden decreased by 43%, and 70% of patients with palpable splenomegaly experienced resolution. Most patients (75%) reported a ≥ 50% reduction in symptom burden, with a median symptom score reduction of 81.5% [51]. No dose-limiting toxicities were observed, and adverse events, predominantly gastrointestinal symptoms such as nausea and diarrhea, were manageable with supportive care. Histopathologic assessments indicated evidence of remission in some patients, and plasma MIC-1 levels showed sustained TP53 activation [51].

In phase II clinical trial (NCT03287245), Idasanutlin was given to 27 patients with HU-resistant or—intolerant PV. At week 32, 56% of patients achieved hematocrit control, including 55% of Ruxolitinib-naive and 60% of Ruxolitinib-resistant/-intolerant patients. Additionally, 50% of patients achieved complete hematologic response, and the overall response rate per modified ELN criteria was 69%. Spleen volume reduction was observed in 92% of patients with baseline splenomegaly, though only 7% achieved a reduction greater than 35%. A rapid and durable reduction in JAK2 V617F allele burden was seen, with a median decrease of 76% by week 32, and bone marrow histopathological remission occurred in 13% of patients. MDM2 inhibitors hold promise as a complementary treatment to standard therapies, offering the potential for enhanced clinical and molecular responses.

Despite these promising outcomes, low-grade gastrointestinal toxicities were frequent, leading to treatment discontinuation in over 50% of participants (See Table 4) [52]. Moreover, it was found to cause a transient TP53 mutant subclones expansion in patient taking Idasanutlin during phase I study [53]. However, further studies are needed to investigate whether the medication induces de novo TP53 mutations, as well as its long-term impact on the management of PV patients.

### Navtemadlin in PV (KRT-232)

After the promising effect of Idasanutlin on PV patients, a new emerging medication KRT-232 Navtemadlin has been investigated. Navtemadlin show a promising efficacy in phase IB/II and Phase III (BOREAS study) among suboptimal response to Ruxolitinib and resistant/ refractory myelofibrosis patients respectively [54, 55]. A phase 1 clinical trial of a 60 mg dose of Navtemadlin (KRT-232) given 4 times at weekly intervals, was safe in healthy subjects. Navtemadlin was well tolerated, with no serious adverse events and minimal impact of food on its absorption. Navtemadlin robustly induced MIC-1, a marker of p53 activation, correlating with drug exposure and confirming effective target engagement [56].

In 2019, KRT-232 was compared with Ruxolitinib in an ongoing randomized, open-label phase 2 trial (NCT03669965) in patients with myelofibrosis who are resistant to or intolerant of HU and/or interferon. Among evaluable patients, spleen volume reductions of ≥ 35% and sustained absence of phlebotomy eligibility were achieved, supporting its potential to meet critical therapeutic goals in PV. In addition to, early reductions in JAK2 V617F allele burden and molecular markers. Treatment-related adverse events (TRAEs), including nausea, diarrhea, fatigue, and anemia, were mostly grade 1 or 2 and manageable [54].

## HDAC inhibitor

### Background and therapeutic rational

Histone acetyltransferases (HATs) and histone deacetylases (HDACs) play key roles in regulating transcriptional events through the reversible acetylation of histones. Aberrant expression of HDACs has been implicated in the development of various tumors [57, 58]. HDAC inhibitors (HDACi) are small molecules that modulate HDAC activity, exerting antiproliferative effects such as promoting cell apoptosis, inducing cell cycle arrest, and promoting differentiation, with minimal impact on healthy cells [58, 59]. HDAC inhibitors have also been shown to enhance the efficacy of JAK2 inhibitors in myeloproliferative disorders by downregulating JAK2 and JAK2 V617F through acetylation of the chaperone protein HSP90 [60]. In vitro, the combination of Givinostat (an HDACi) and HU induced a synergistic cytotoxic effect in JAK2V617F-positive cell lines, resulting in 56.6% cell death in HEL cells (P < 0.05) and 75.3% in UKE1 cells, significantly exceeding the additive expectations [61].

### Givinostat in PV (ITF2357)

In phase I clinical trial, single doses (50–600 mg) and multiple doses (50–200 mg/day) were well-tolerated, with mild-to-moderate adverse events such as headache, nausea, and palpitations, primarily at higher doses. Platelet counts decreased in a dose-dependent manner but normalized within weeks after treatment cessation. Pharmacokinetic analyses showed dose-proportional absorption with a half-life of 5–7 h, supporting a twice-daily dosing regimen [62].

In Phase IIA clinical trial (NCT00606307), Givinostat was evaluated in treating PV and other myeloproliferative neoplasms (MPNs) with the JAK2V617F mutation. In PV/essential thrombocythemia (ET) patients, Givinostat demonstrated clinical efficacy, achieving a complete or partial response in 58% of cases. Key benefits included a reduction in phlebotomy requirements, a decrease in spleen size in 75% of patients with splenomegaly, and a significant reduction in pruritus, a common symptom in PV. Although molecular responses (reduction in the JAK2V617F allele burden) were observed, complete molecular remission was not achieved. The drug was generally well tolerated, with gastrointestinal symptoms like diarrhea and nausea being the most common side effects [63].

Both the Phase Ib/II trial (NCT01901432) and the long-term follow-up trial (NCT01761968) of Givinostat in PV showed high efficacy, with over 70% of patients achieving an overall response in the Phase Ib/II trial and sustained response rates exceeding 80% in the long-term study. Both trials demonstrated significant improvements in hematologic parameters, including normalization of hematocrit, white blood cell, and platelet counts, as well as reductions in JAK2V617F allele burden and spleen size (See Table 3). Symptom control, including pruritus resolution, was notable in the Phase Ib/II trial, and disease progression was infrequent in the long-term study, with only 2.8 cases of progression per year. Regarding safety, adverse events were mostly mild to moderate in the Phase Ib/II trial, with thrombocytopenia and diarrhea being common. In the long-term trial, however, 10% of patients experienced grade 3 side effects, including thrombocytopenia and QTc prolongation [64]. Both trials confirm givinostat’s effectiveness in PV, with long-term data supporting its durability but emphasizing the need to monitor for severe adverse events.

In a phase II multicenter (NCT00928707), open-label study that evaluated the efficacy and safety of Givinostat, in combination with hydroxycarbamide (HC) in 44 PV patients unresponsive to maximum tolerated doses of HC monotherapy. After 12 weeks of treatment, a complete or partial response was observed in 55% of patients receiving 50 mg of Givinostat and 50% of those receiving 100 mg, with 42% of previously phlebotomy-dependent patients achieving phlebotomy independence. Notably, 64%-67% of patients experienced resolution of pruritus, a symptom that significantly impacts quality of life in PV. The treatment was generally well tolerated, with grade 3 adverse events observed in only two patients (4.5%) across both dose levels. At 24 weeks, 50% of non-responders from the 50 mg group who had their dose increased to 100 mg achieved partial response [65]. A phase III, multicenter trial (NCT06093672) is comparing efficacy and safety of Givinostat to HU in Jak2V617F-positive high-risk PV patients [66] (see Table 3).

## LSD1 inhibitor

### Background and study rational

Lysine-specific demethylase 1 (LSD1) is a histone demethylase enzyme that plays a crucial role in regulating the self-renewal of hematopoietic stem and tumor-initiating cells [67]. Aberrant overexpression of LSD1 drives multiple cancer hallmarks, including myeloproliferative neoplasms (MPNs), by enhancing proliferation, metastasis, angiogenesis, and disrupting cell cycle progression [68, 69]. Bomedemstat is a small-molecule LSD1 inhibitor (IMG-7289) that has been tested in various hematopoietic cancers, including essential thrombocythemia (ET), AML, MF, and PV [68]. Bomedemstat appears to have a selective effect, inhibiting proliferation and inducing apoptosis by downregulating the anti-apoptotic protein BCLXL and upregulating p53 [70]. In 89 MPN patients, Bomedemstat lead to improvement in symptoms in 89%, hematological stability.

in 86%, reduction in spleen size in 78%, stabilization or reduction in bone marrow in 83% and decrease of mutant allele burden in 42% along with acceptable safety profile [71]. In a phase IIb trial on essential thrombocythemia patients, Bomedemstat demonstrated a significant reduction in platelet count without affecting hemoglobin levels and reduced disease burden. However, 12 serious adverse events were reported [72].

### Bomedemstat (IMG-7289)

A phase II clinical trial (NCT05558696) is currently underway to evaluate the safety, efficacy, pharmacokinetics (PK), and pharmacodynamics of the orally administered bomedemstat, in participants with PV [73]. A second phase II trial (NCT04262141) is ongoing to assess the effect of Bomedemstat in PV and ET patients that failed at least on standard therapy and expected to complete by 2025 [74].

## Limitations

The novel therapies have several limitations that hinder their applicability and raise concerns about clinical efficacy. Across trials in different phases, sample sizes were small, resulting in limited statistical power—for example, the Phase II bomedemstat studies (NCT04262141 and NCT05558696) enrolled only 4 and 20 patients, respectively [73, 74]. A second limitation is the uncertain safety profile of some agents. The clinical applicability of the MDM2 inhibitor idasanutlin was curtailed by a high discontinuation rate, compromising its benefit–risk profile; 41% of patients discontinued before 32 weeks, predominantly because of low-grade gastrointestinal toxicities [52]. In addition, idasanutlin has been associated with expansion of pre-existing TP53 mutations, and the emergence of de novo TP53 mutations cannot be excluded; supporting this association, most TP53-mutant subclones declined after idasanutlin discontinuation [53]. Givinostat was also linked to severe adverse events, including thrombocytopenia and QTc prolongation, despite general tolerability in most participants during Phase II trials, underscoring the need for longer follow-up and close clinical monitoring [64].

Beyond these agent-specific issues, long-term follow-up data and evidence for the durability of molecular remissions remain limited across programs. Notably, while virtually all recent trials specified hematocrit control or complete/partial response as the primary endpoint and time to response and adverse events as secondary endpoints none assessed efficacy in terms of reducing thrombotic risk in patients with PV or progression to myelofibrosis or leukemia. Moreover, not all the recent studies systematically evaluate molecular response (e.g., reduction in JAK2 V617F allele burden), with the exception of the Idasanutlin (NCT02407080, NCT03287245), and Givinostat (NCT01761968) trials.

## Conclusion

In conclusion, the management of polycythemia vera is rapidly advancing with the development of targeted therapies that aim not only to control hematocrit levels but also to alleviate debilitating symptoms and address the underlying disease mechanisms. Novel therapies such as hepcidin agonists, MDM2 inhibitors, histone deacetylase (HDAC) inhibitors, and LSD1 inhibitors have shown promising results in preclinical and early-phase clinical trials, offering hope for more effective and less toxic treatment options. However, challenges remain in determining the long-term efficacy, safety, and optimal treatment regimens for these therapies. Most of these novel medications have been associated with common adverse effects such as gastrointestinal disturbances, fatigue, and hematologic toxicity, which need to be carefully monitored. Future research, including larger clinical trials and studies that assess patient-reported outcomes, is crucial for refining these therapies and understanding their broader impact on patient quality of life. As our understanding of the molecular mechanisms behind PV continues to grow, these emerging therapies hold the potential to significantly improve clinical management, offering patients a more individualized and comprehensive approach to treatment.

## Data Availability

No datasets were generated or analysed during the current study.
